# Function of the ERFL1a Transcription Factor in Wheat Responses to Water Deficiency

**DOI:** 10.3390/ijms19051465

**Published:** 2018-05-15

**Authors:** Tian Gao, Ge-Zi Li, Chuan-Ren Wang, Jie Dong, Sha-Sha Yuan, Yong-Hua Wang, Guo-Zhang Kang

**Affiliations:** 1 The National Key Laboratory of Wheat and Maize Crop Science, Henan Agricultural University, Zhengzhou 450002, China; gaotian1211@henau.edu.cn (T.G.); wangchuanren@henau.edu.cn (C.-R.W.); 2 The Collaborative Center Innovation of Henan Food Crops, Henan Agricultural University, Zhengzhou 450002, China; dongjie@henau.edu.cn (J.D.); shashayuan@henau.edu.cn (S.-S.Y.); 3 The National Engineering Research Center for Wheat, Henan Agricultural University, Zhengzhou 450002, China; ligezi@henau.edu.cn (G.-Z.L.); wangyonghua88@henau.edu.cn (Y.-H.W.)

**Keywords:** TaERFL1a, *Triticum aestivum* L., abiotic stress, BSMV-VIGS, Yeast two-hybrid

## Abstract

The APETALA2/ethylene response factor (AP2/ERF) superfamily is involved in the responses of plants to biotic and abiotic stresses; however, the functions and mechanisms of some members of this family in plants are unclear. In our previous study, expression of *TaERFL1a*, a member of the AP2/ERF family, was remarkably induced in wheat seedlings suffering freezing stress. In this study, we show that its expression was rapidly upregulated in response to salt, cold, and water deficiency, suggesting roles in the responses to abiotic stresses. Further, transient barley stripe mosaic virus-induced gene silencing (BSMV-VIGS) resulted in significantly reduced tolerance to 20% PEG6000-stimulated water deficiency. Subcellular localization and transcriptional activation assays separately showed that TaERFL1a was targeted to the nucleus and possessed transcriptional activation activity. Yeast two-hybrid library screening identified six interacting proteins, and of these, the interactions between TaERFL1a and TaSGT1, and TaERFL1a and TaDAD2 proteins were further confirmed by yeast co-transformation and bimolecular fluorescent complementation (BiFC). Collectively, our results suggest that TaERFL1a is a stress-responsive transcription factor, which could be functionally related to proteins involved in the abiotic stress responses of plants.

## 1. Introduction

In plant growth, development, and responses to biotic and abiotic stresses, transcription factors regulate the expression of multiple downstream target genes by binding to *cis*-acting elements in the promoters [1]. Recent studies have identified several major superfamilies of transcription factors, including APETALA2/ethylene response factor (AP2/ERF), basic leucine zipper (bZIP), Cys2(C2)His2(H2)-type zinc fingers (ZFs), myeloblastosis (MYB) oncogene, and WRKY [2]. The AP2/ERF superfamily is characterized by the presence of an AP2/ERF DNA-binding domain of 60–70 amino acids, and is composed of the ERF, AP2, and RAV (RELATED TO ABI3 (ABASCISIC ACID INSENSITIVE3) and VP1 (VIVIPAROUS1)) families. ERF and AP2 family proteins contain one and two AP2/ERF domains, respectively, while RAV family proteins also have one AP2/ERF domain as well as a B3 domain, which is a DNA-binding domain conserved in other plant-specific transcription factors, including VP1/ABI3 [3].

The three AP2/ERF families play distinct roles in higher plants [4]. AP2 family proteins mainly participate in the regulation of developmental processes, e.g., flower and embryo development [5]. Members of the RAV family are reportedly involved in responses to ethylene and brassinosteroid [6]. Many members of the ERF family are both biotic/abiotic stress-responsive and bind the GCC-box or DRE/CRT elements in the promoter of downstream target genes [7,8]. Based on the conserved domains, the ERF subfamily in plants is further divided into subgroups B1 to B6 [9]. Despite their common function in transcription activation, some B1 ERFs reportedly repress the transcription of their target genes and some other transcription factors, whereas the B2 and B3 subgroups induce target gene transcription and enhance tolerance to environmental stresses [10]. The various ERF members may play distinct roles in plant responses to the same environmental stress, or the same ERF members may have diverse functions in the responses to different environmental stresses [11]. For example, ERF2 and ERF4 reportedly positively and negatively, respectively, regulate the response to cold [4,12]. The tomato ERF gene *TSRF1* enhances pathogen resistance and drought tolerance, but suppresses the response to osmotic stress [13,14].

Xu and colleagues used a conserved ERF domain as a probe to screen a drought-induced wheat cDNA library, and isolated two wheat ERF members—an ethylene-responsive factor (TaERF1) and an ethylene-responsive factor-like (TaERFL1a) (GenBank accession numbers: AY781352 and BQ789086, respectively) [15]. Expression of *TaERF1* was significantly induced by drought, salt, cold, abscisic acid, ethylene, salicylic acid, and pathogen (*Blumeria graminis*), and its heterologous expression in Arabidopsis plants improved their resistance to pathogens (*Botrytis cinerea* and *Pseudomonas syringae*) and abiotic stresses (drought, cold, and salt) [15]. To our knowledge, however, the function of the TaERFL1a transcription factor has not yet been reported.

Our previous study involved global transcriptional profiling using Affymetrix Wheat GeneChip microarray analysis of wheat seedlings suffering from −5 °C freeze stress. We identified 102 genes whose transcript levels changed at least eight-fold after 1 day or 3 days of freeze stress. Of these freeze-responsive genes, the transcript levels of a probe (probe set ID: Ta.14000.1.S1_-_at) increased by 16.27- and 9.46-fold after 1 and 3 days of freeze stress, respectively. This probe was matched with TaERFL1a [16], suggesting that it could function in the response to freeze stress. In this study, its expression under other abiotic stresses (e.g., cold, salt, and water-deficiency) was measured by quantitative real-time PCR (qPCR), its role in abiotic tolerance was evaluated by the barley stripe mosaic virus-induced gene silencing (BSMV-VIGS) method, its subcellular localization in tobacco leaf was visualized using green fluorescent protein, and its transcriptional activation activity and two interacting proteins were identified using a yeast two-hybrid library and further verified by yeast co-transformation and BiFC assay. Our results suggest TaERFL1a is a novel member of the ERF family, which functions as a transcriptional activator and positively regulates the response of wheat to water-deficiency stress.

## 2. Results and Discussion

### 2.1. Identification of TaERFL1a

**Figure 1:**
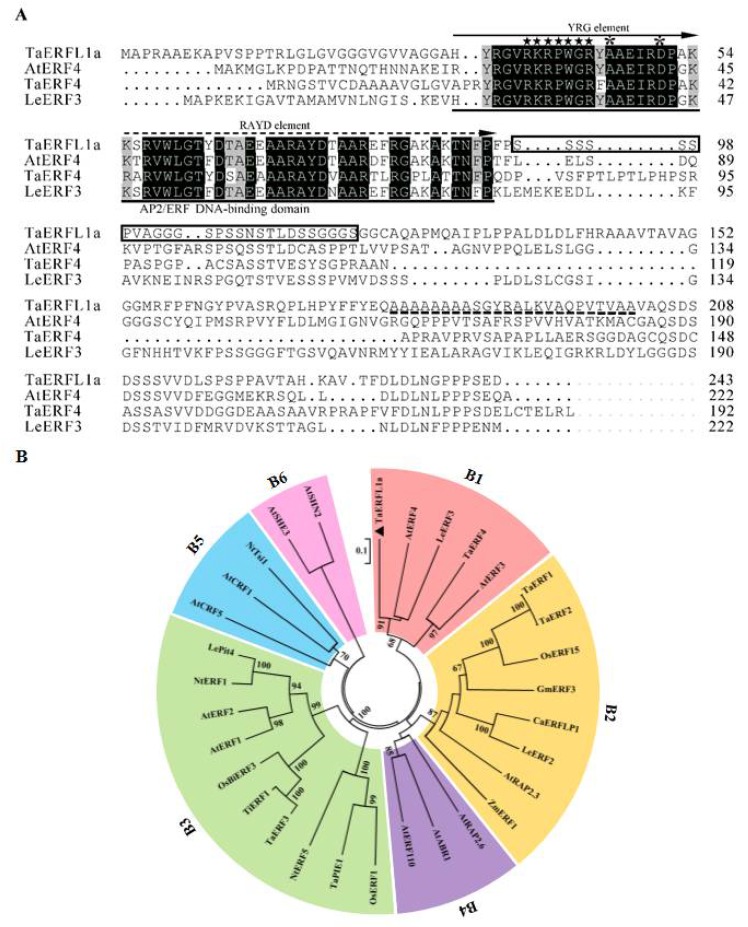
Multiple alignment (**A**); and phylogenetic tree (**B**) of TaERFL1a. Multiple alignment of TaERFL1a with AtERF4 (AY140030), TaERF4 (JX014257), and LeERF3 (AY192369). Boxes in black and gray shades represent 100.0% and 75.0% similarities of ERF proteins in B1 subgroup, respectively. AP2/ERF DNA binding domain is indicated with single line, and it contains conserved YRG (YRGVRKRPWGRYAAEIRDPAK) and RAYD (KSRVWLGTYDTAEEAARAYDTAAREFRGAK AKTNFP) elements and they are indicated with solid and dotted arrows, respectively. The nuclear localization signal sites are shown in pentagrams. The presence of 14 th amino acid (A) and 19th amino acid (D) in ERF subfamily are signed with two stars. The serine-rich and alanine-rich regions are marked by boxes and dotted lines, respectively. Phylogenetic analysis of AP2/ERF transcription factors in *Arabidopsis thaliana*, *Lycopersicon esculentum*, *Oryza sativa*, *Glycine max*, *Capsium annum*, *Nicotiana tobacum*, *Triticum aestivum*, and *Thinopyrum intermedium* plant species. Six subgroups are presented in different colors. The phylogenetic tree was constructed using neighbor-joining phylogeny of MEGA 5.0 with default parameter.

Bread wheat (*Triticum aestivum* L.) is an allohexaploid species containing three closely related, yet distinct, subgenomes (A, B, and D), which contain complementary sets of homologous genes that share more than 95% sequence identity in their coding regions. Thus, the majority of wheat genes are present as three copies among the A, B, and D subgenomes [17,18]. Transcriptomic analysis has shown that, in developing grains of allohexaploid wheat, one homolog of most genes is active only in one subgenome, while the other homologs in the other two subgenomes are either eliminated or partially or completely suppressed through genetic or epigenetic modifications (genomic asymmetry or expression bias) [19,20]. Under environmental stresses (drought, heat, and pathogen), a large proportion (≥63.9%) of wheat homologs also exhibited expression bias [21,22]. *TaERFL1a* (GenBank accession number: BQ789086) was first identified in a drought-induced wheat cDNA library, and in our previous study, its transcript levels were shown to be sharply upregulated in freeze-stressed wheat seedlings [16]. The cDNA sequence of *TaERFL1a* from the wheat cultivar Yumai 34 constitutes a complete open reading frame of 732 bp encoding a putative protein of 243 amino acids (Figure 1A), which has a high level of similarity (96.1%) to BQ789086 (Figure S1). BLAST searching against the International Wheat Genome Sequencing Consortium (IWGSC) wheat genome database showed that *TaERFL1a* has a high level of similarity (100.0%) to a chromosome-located contig, TRIAE_CS42_3B_TGACv1_222710_AA0769340. Transcripts of the other two TaERFL1a homologs (TRIAE_CS42_3AL_TGACv1_194146_AA0627440 and TRIAE_CS42_3DL_TGACv1_252924_AA0892640) were not found in freeze-stressed wheat seedlings in our previous study [16]. Moreover, their coding regions were not truncated (premature termination codon or splice acceptor/donor site) (Figure S2), possibly because they undergo post-transcriptional regulation, e.g., alternative splicing and/or RNA processing [19,20].

Similar to the structures of other ERFs [23], the predicted TaERFL1a protein had an AP2/ERF domain containing a nuclear localization signal (KRPWGR), an alanine-rich region (AAAAAAAASGYRALKVAQPVTVAA), and a serine-rich region (SSSSSSPVAGGGSPSSNSTLDS SGGGS) (Figure 1A). Phylogenetic analysis indicated that the AP2/ERF domain of TaERFL1a shares more than 91.4% identity with those of AtERF4 and LeERF3, and that it belongs to one subbranch of the B1 subgroup in the ERF subfamily, implying that these three ERF proteins could have similar biological functions. However, the AP2/ERF domain of TaERFL1a had a low (<40.7%) level of similarity with other wheat ERF proteins (Figure 1B). Therefore, TaERFL1a may be a novel wheat ERF protein, potentially with a new function.

In the B1 subbranch, AtERF4 and LeERF3 act as transcriptional repressors, negatively regulating the iron deficiency response in *Arabidopsis thaliana* and fruit ripening in tomato, respectively [24,25]. To our knowledge, however, their abiotic tolerance function has not been reported to date.

### 2.2. Expression Profiles of TaERFL1a during the Responses of Wheat to Abiotic Stresses and Abscisic Acid

**Figure 2:**
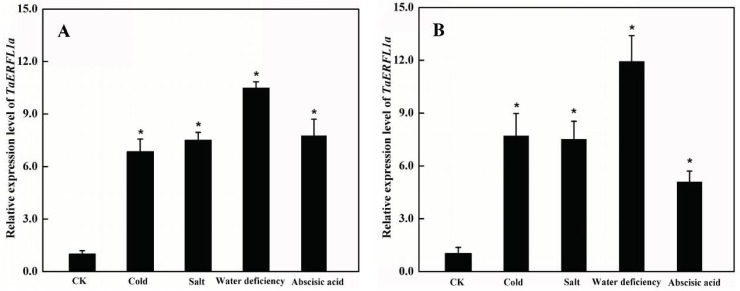
Transcript levels of *TaERFL1a* in leaves of wheat plants suffering from cold (4 °C), salt (250 mM NaCl), water deficiency (20% PEG6000), and abscisic acid (0.1 mM) for 3 days. Transcript levels are determined by qPCR using *Actin* (**A**) and *GAPGH* (**B**) genes as internal controls. Each value is mean ± standard deviation of three biological replicates. Asterisks indicate significant differences (*p* < 0.05).

In view of its potential role in the response to freeze stress [16], we determined the transcript levels of *TaERFL1a* by qPCR in wheat exposed to cold (4 °C), salt (250 mM NaCl), and water deficiency (20% PEG6000) stresses and abscisic acid. During the ~220 days annual growing period in China, wheat plants often suffer these abiotic stresses [26]. Using the wheat *Actin* gene as the internal control, the qPCR data indicated that, at 3 days after cold, salt, water deficiency, and abscisic acid, *TaERFL1a* transcript levels had increased 6.86-, 7.51-, 10.45- and 7.80-fold, respectively (Figure 2A). Similar data were obtained using the *glyceraldehyde 3-phosphate dehydrogenase* (*GAPDH*) gene as the internal control (Figure 2B). Therefore, *TaERFL1a* may be involved in the responses of wheat to diverse abiotic stresses, and, similar to in pathogen resistance [27], it may be one member of abscisic acid signaling pathway.

### 2.3. Function of TaERFL1a in the Response of Wheat to Abiotic Stresses

Functional characterization of genes in higher plants can be performed by transgenic and mutational approaches. However, the low transformation efficiency, multiple copy insertions, cultivar-specificity, time-consumption, and high cost of transgenic approaches in bread wheat have limited the progress in functional genomics in this species. Moreover, it is difficult to generate null mutants because of the functional redundancy of homologous genes in this species [18]. Therefore, functional characterization of bread wheat genes has lagged compared to other important crops, such as maize and rice. BSMV-VIGS facilitates rapid generation of gene knockdown phenotypes in polyploid plants, because plant transformation is not required, which accelerates characterization of target genes [28].

To verify the function of TaERFL1a in the response of wheat to abiotic stresses, we inoculated a BSMV-VIGS-TaERFL1a-silencing vector into the second fully developed leaves of three-leaf stage wheat plants (see Materials and Methods). BSMV-VIGS-GFP-inoculated wheat plants were used as the control. At 8 d after inoculation, wheat seedlings inoculated with the BSMV-VIGS-TaERFL1a silencing vector or BSMV-VIGS-GFP vector exhibited visible photobleaching symptoms (Figure S3A), and the transcript levels of *TaERFL1a* in BSMV-VIGS-TaERFL1a-inoculated wheat seedlings was decreased by 79.0% (Figure S3B,C). Therefore, the *TaERFL1a* gene was successfully silenced in wheat seedlings.

**Figure 3:**
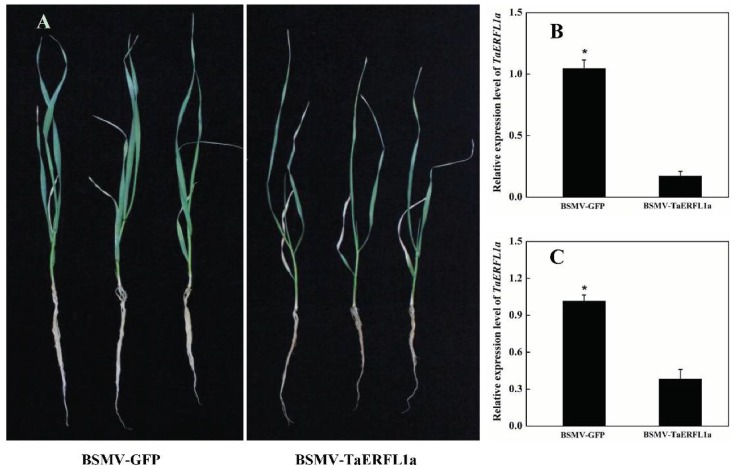
Phenotypes of BSMV-TaERFL1a-inoculated and BSMV-GFP-inoculated wheat seedlings suffering from 20% PEG6000 for 3 d (**A**); and transcript levels of *TaERFL1a* gene at this sampling timepoint (**B**,**C**). Transcript levels of *TaERFL1a* gene were measured by qPCR using *Actin* (**B**) and *GAPDH* (**C**) genes as internal control. Each value is mean ± standard deviation of three independent biological replicates. Asterisks indicate significant differences (*p* < 0.05).

Subsequently, BSMV-VIGS-TaERFL1a- and BSMV-VIGS-GFP-inoculated wheat seedlings (control) were separately transferred to Hoagland solution supplemented with 20% PEG6000 to be subjected to water deficiency for 3 d. Compared to BSMV-VIGS-GFP-inoculated wheat seedlings, BSMV-VIGS-TaERFL1a-inoculated wheat seedlings exhibited significant and deleterious phenotypes after 3 d of stress such as curled and wilted leaves and inhibited growth (Figure 3A). These qualitative phenotypic effects were confirmed by quantitative analysis of plant fresh and dry weights (Table 1). In addition, the absolute water contents of the BSMV-VIGS-TaERFL1a-inoculated wheat seedlings were significantly lower than that of the BSMV-VIGS-GFP-inoculated wheat seedlings. However, the concentrations of malondialdehyde (MDA), a common product of lipid peroxidation and a sensitive diagnostic indicator of oxidative injury, was significantly increased in the BSMV-VIGS-TaERFL1a-inoculated wheat seedlings (Table 1). At this time, the *TaERFL1a* transcript levels were significantly reduced in BSMV-VIGS-TaERFL1a-inoculated wheat seedlings (Figure 3B,C), implying that expression of this gene is inhibited during water deficiency. Therefore, BSMV-VIGS-TaERFL1a-inoculated wheat seedlings were more sensitive to water deficiency than control seedlings, and unlike the negative role played by AtERF4 in the response to Fe deficiency [24], TaERFL1a may enhance the response of wheat to water deficiency. Like in PEG-stimulated water deficiency, the significantly improved transcription levels regulation of TaERFL1a significantly increased under cold and salt (Figure 2) also implied that it may also confer the tolerance to cold and salt stresses.

### 2.4. Subcellular Localization of TaERFL1a

**Figure 4:**
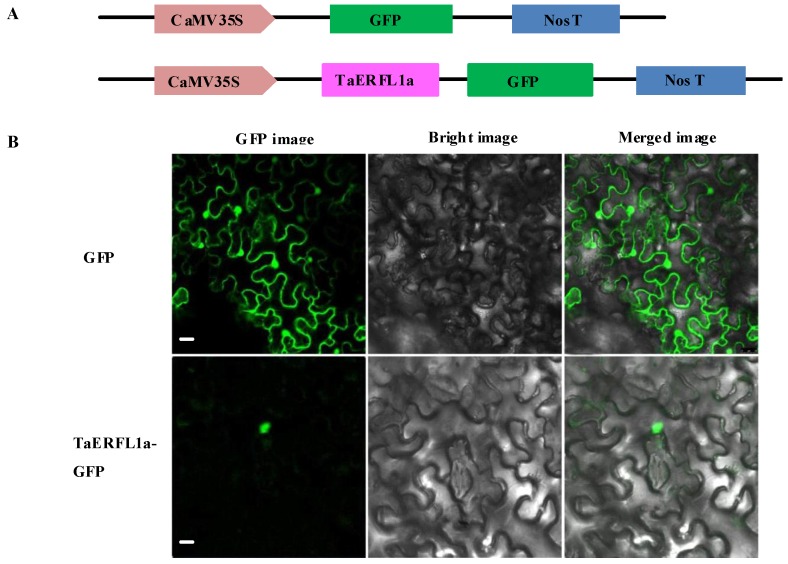
Subcellular localization of TaERFL1a protein in tobacco leaves: (**A**) structure of TaERFL1a-GFP fusion expression vector; and (**B**) subcellular localization of single GFP protein (GFP), and TaERFL1a-GFP fusion protein (TaERFL1a-GFP) in tobacco leaves. Bar = 20 μm.

If TaERFL1a functions as a transcription factor, it should be localized to the nucleus. The coding sequence of *TaERFL1a* was fused with that of GFP under the control of the *Cauliflower mosaic virus* (35S) promoter to generate a construct expressing a TaERFL1a-GFP fusion protein (Figure 4A). This construct was delivered into tobacco leaves using an *Agrobacterium*-mediated transient expression method. GFP fluorescence was detected only in the nucleus (Figure 4B), indicating that TaERFL1a is localized to the nucleus and could function as a transcription factor.

### 2.5. Interacting Proteins of TaERFL1a

**Figure 5:**
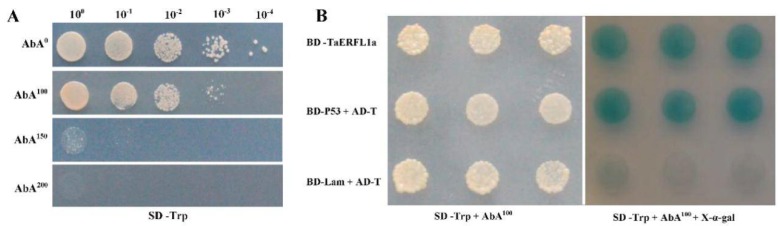
Growth of yeast strain Y2HGold containing pGBKT7-TaERFL1a on the selective media with different concentrations of AbA (**A**); and transcription activation of TaERFL1a (**B**). Concentrations of AbA are used at 0, 100, 150, and 200 ng/mL in Y2H assay. Transformed yeast cells harboring TaERFL1a grow on SD/-Trp + AbA^100^, or SD/-Trp + AbA^100^ + X-α-gal media. BD-P53 + AD-T (pGBKT7-P53 + pGADT7-RecT) and BD-Lam + AD-T (pGBKT7-Lam + pGADT7-RecT) act as the positive and negative controls, respectively.

The yeast two-hybrid (Y2H) assay enables study of protein–protein interactions and is widely used to assess the biological functions of target proteins and to identify novel proteins [29,30]. In this study, the bait protein expression plasmid pGBKT7-TaERFL1a and a wheat leaf cDNA library (prey vector) were constructed, and the GAL4 DNA-binding domain was fused to the full-length of TaERFL1a (see Materials and Methods). TaERFL1a was autoactivated at 30 °C for 5 d in the Y2H Gold yeast strain in SD/–Trp medium with 0, 100, 150, or 200 ng/mL aureobasidin A (AbA) (Figure 5A). Yeast growth decreased with increasing AbA concentration, and no yeast clone was produced in the presence of 200 ng/mL AbA (Figure 5A). At 100 ng/mL AbA, yeast cells expressing TaERFL1a grew well and formed blue colonies on SD/–Trp medium, whereas the negative control did not (pGBKT7-Lam) (Figure 5B). Therefore, TaERFL1a has transcriptional activation activity, and could function as a transcriptional activator of target genes.

**Figure 6:**
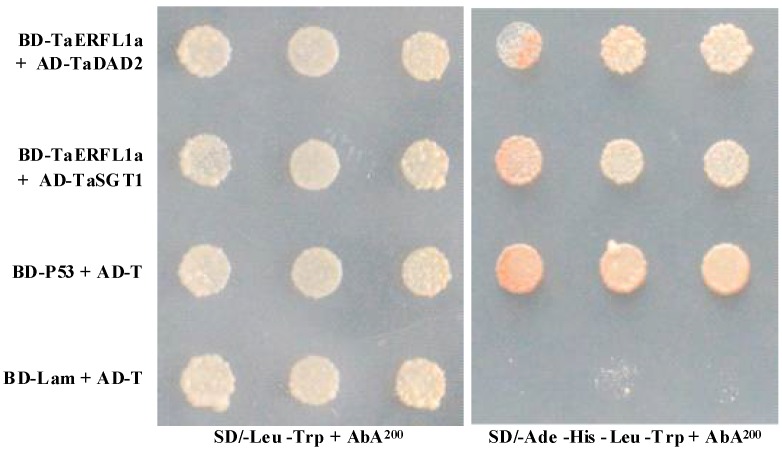
Interactions of TaERFL1a and TaSGT1, or TaERFL1a and TaDAD2 in a Y2H system. Transformed TaERFL1a and TaSGT1, or TaERFL1a and TaDAD2 grow on SD/-Leu/-Trp and SD/-Ade-His-Leu-Trp + AbA^200^ selected media. BD-P53 + AD-T (pGBKT7-P53 + pGADT7-RecT) and BD-Lam + AD-T (pGBKT7-Lam + pGADT7-RecT) represent the positive and negative controls, respectively.

We tested TaERFL1a interaction with proteins encoded by a wheat leaf cDNA library by Y2H screening resulting in the identification of 10 colonies (Table 2), implying their interaction with TaERFL1a. Sequencing of these colonies resulted in identification of six genes (proteins), two of which were each detected three times (Table 2). The proteins encoded by the six genes were a Cu/Zn superoxide dismutase (Cu/Zn SOD, one clone), a suppressor of the G2 allele of S-phase kinase-associated protein 1 (TaSGT1, one clone), a defender against cell death 2 (TaDAD2, one clone), a pyrrolidone-carboxylate peptidase (one clone), an avenin-like a precursor (3 clones), and an MOB kinase activator-like (three clones) (Table 2). Similar to in *TaERFL1a* (Figure 2), the transcription levels of most of the genes encoding these identified proteins were also enhanced in wheat responses to three similar abiotic stresses (4 °C, 250 mM NaCl, and 20% PEG6000), whereas their transcript levels were remarkably inhibited in BSMV-VIGS-TaERFL1a-inoculated wheat seedlings at 8 days after virus inoculation (Figure S4). These also implied that these six proteins could be regulated by TaERFL1a. Co-transformation of the bait vector (TaERFL1a in pGBKT7) together with the individual prey vector of the identified interactors (TaSGT1 or TaDAD2 in pGADT7) into Y2H Gold yeast confirmed the above interactions (Figure 6).

**Figure 7:**
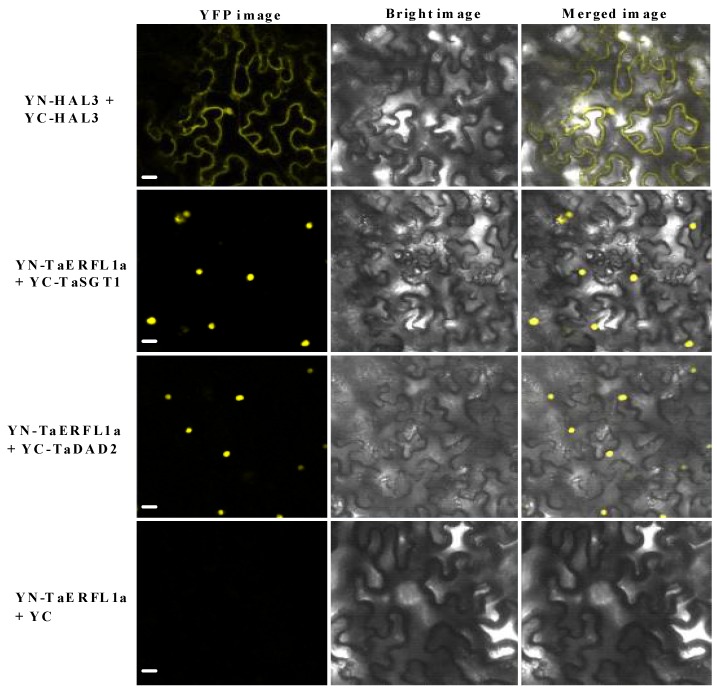
BiFC assay of the interaction between TaERFL1a and TaSGT1, or between TaERFL1a and TaDAD2 proteins in tobacco leaves. The YN-TaERFL1a and YC-TaSGT1 or YC-TaDAD2 constructs were co-infiltrated in tobacco. The YFP fluorescence was detected by confocal laser scanning microscopy. Co-transformants of YN-HAL3 and YC-HAL3 as well as YN-TaERFL1a and YC were used as positive and negative controls, respectively. Bar = 20 μm.

If there were the interactions between transcription factors and other proteins, these proteins should be located at the nucleus. Thus, to confirm these interactions, subcellular localization assay on both TaSGT1 and TaDAD2 were further performed and our results showed that these two proteins were located at the nucleus and cytoplasm (Figure S5), indirectly implying that they could interact with TaERFL1a transcription factor. In this study, moreover, BiFC experiments between TaERFL1a and TaSGT1, or between TaERFL1a and TaDAD2 were assayed and our data indicated that TaERFL1a interacted with these two proteins (Figure 7), further confirming their interactions. The functions of these two proteins are discussed below.

The 90-kDa heat shock protein (HSP90) is an ATP-dependent molecular chaperone, which is associated with maintaining and regulating the conformation of many proteins, and with protecting normal cells from stress stimuli [31]. SGT1 has been found to be one important co-chaperone of HSP90s and they form protein complexes. Previous studies have found that SGT1 plays an important role as a resistance-related gene and is induced during various biotic stress conditions in plants [32]. Similarly, *DAD2* gene has also been characterized to function as a suppressor of cell death in the early stages of wheat–stripe rust fungus interaction [33]. To our knowledge, however, functions of SGT1 and DAD2 proteins in abiotic stresses have not been identified in higher plants. In *Brassica oleracea*, Shanmugam and his colleagues found that the expression levels of two *SGT1* genes (*BolSGT1a* and *BolSGT1b*) were significantly induced in response to heat, cold, drought, and salt, and they are strongly associated with co-regulators (HSP90, etc.) during stress conditions, implying that they could be potential target genes in response to abiotic stresses [34]. Several plant ERF transcription factors bind to the promoters of downstream target genes that have the GCC-box (AGCCGCC) and/or DRE/CRT (G/ACCGAC) elements, which are abiotic and biotic stress-responsive [35], the promoter of the *TaSGT1* gene also contained the GCC-box and DRE/CRT elements (Figure S6), suggesting that TaERFL1a could interact with TaSGT1 via these two elements. Its interactions with TaSGT1 and TaDAD2 suggest that TaERFL1a could be a common regulator in the responses of plants to diverse biotic and abiotic stresses.

Interactions between TaERFL1a and other four identified proteins were not determined, and, thus, there might be false interactions between TaERFL1a and these four proteins. For example, TaSOD identified in our study, is located at cytoplasm, not in the nucleus [36], and it could not be directly interacted with TaERFL1a. However, TaSOD could be combined with other proteins, which could interact with TaERFL1a, to form a protein complex [37]. Thus, TaSOD could be indirectly regulated by TaERFL1a.

## 3. Materials and Methods

### 3.1. Identification and Sequence Analysis of TaERFL1a

In our previous study, 5-leaf wheat seedlings (at the floret primordium differentiating stage of spike development) of bread wheat (*Triticum aestivum* L. Yumai 34 cultivar) were subjected to −5 °C freeze stress for 3 days, and the uppermost fully expanded leaves were collected from each cultivar at 0, 1, and 3 days after freeze stress for cDNA microarray analysis [16]. Among the identified freeze-responsive genes, a probe (probe set ID: Ta.14000.1.S1_-_at) was induced 16.27- and 9.46-fold after 1 and 3 days of freeze stress, respectively, and matched TaERFL1a (GenBank accession number: BQ789086). In this study, the coding region of *TaERFL1a* was amplified from cDNA extracted from Yumai 34 leaves using primers based on the nucleotide sequence of *TaERFL1a* (BQ789086). The amplified sequence was searched against the IWGSC wheat genome database (https://www.wheatgenome.org/). The amino acid sequence similarities of plant ERFL1a proteins were determined using BLASTX and the NCBI database (http://www.ncbi.nlm.nih.gov/BLAST/). Plant ERFL1a amino acid sequences were retrieved from the GenBank database, and a phylogenetic tree was constructed using MEGA software 5.0 (Kumar, S Tempe, AZ, USA) with the ClustalW method. Sequence alignments were conducted using DNAMAN software (Lynnon Biosoft, San Ramon, CA, USA).

### 3.2. Plant Materials and Abiotic Stresses

Seeds of bread wheat (Yumai 34 cultivar) were sterilized with 0.01% HgCl_2_ and washed at least three times with distilled water. The sterilized seeds were hydroponically grown in glass dishes (15 cm diameter, 60 seedlings per dish) in full-strength Hoagland solution [38]. Germinated seedlings were transferred to an FPG-300C-30D light incubator (NingBo Technology Co., Ltd., NingBo, China) under a 16/8-h (light/dark, 25/15 °C) photoperiod, light intensity of 250 μmol m^−2^ s^−1^, and relative humidity of 60/75% (day/night). After 12 days, wheat plants at the 3-leaf stage, at which plants are in the autotrophic stage and are sensitive to abiotic stresses [39], were subjected to cold (4 °C), salt (250 mM NaCl), or water deficiency (20% PEG6000) stress, or abscisic acid (0.1 mM) for 3 days. The uppermost fully expanded leaves were harvested, frozen in liquid nitrogen, and stored at −80 °C until required.

### 3.3. Transcript Level of TaERFL1a

Total RNA was extracted from wheat leaves using TRIzol RNA Isolation Reagent (Invitrogen, Carlsbad, CA, USA) and treated with RNA-free DNase I (TaKaRa Biotechnology (Dalian) Co., Ltd., Dalian, China). Total RNA extraction, first-strand complementary cDNA synthesis, and qPCR were performed as described previously [40]. The primers used are listed in Table S1. Relative transcript levels of *TaERFL1a* were calculated using the 2^−ΔΔ*C*t^ method, with wheat *Actin* and *GAPDH* (GenBank accession Nos. AB181991 and EF592180) as the internal controls.

### 3.4. Transient Silencing of TaERFL1a

The functional complementation among homologs in the allohexaploid bread wheat genome hampers generation of a complete null cross [18]. To prevent functional complementation and allow complete silencing, we selected a conserved cDNA fragment (239 bp, +305 to +543 bp, translation start ATG is +1) of TaERFL1a, the three copies of which shared 98.4% similarities (Figure S2), possibly enabling simultaneous silencing by BSMV-VIGS. In addition, the selected fragment was outside the AP2/ERF domain to avoid silencing of other members of the ERF family (Figure S2). The primers and a schematic of the BSMV-VIGS-TaERFL1a vector are shown in Table S1 and Figure S7, respectively. Viral vector construction, viral RNA transcription, and viral inoculation for BSMV-VIGS were performed as described previously [41,42]. BSMV-VIGS-GFP-inoculated wheat seedlings were used as the negative control, and the appearance of chlorosis on the inoculated tissues confirmed successful BSMV inoculation and target gene silencing in the host plant [43].

The RNA-*α*, RNA-*β*, and RNA-*γ* transcripts of the BSMV-VIGS-TaERFL1a or BSMV-VIGS-GFP vectors were mixed in a 1:1:1 ratio and diluted with nine volumes of diethyl pyrocarbonate (DEPC)-treated water. Next, 12 volumes of 2× GKP buffer (50 mM glycine, 30 mM, pH 9.2 dipotassium hydrogen phosphate, 1% bentonite, 1% Celite) were added. Virus inoculation was performed on the second leaves of wheat seedlings (Yumai 34 cultivar), and was accomplished by gently rubbing the leaf surface five times from the central section of wheat leaves to the tip and then from base to tip [42]. At 8 days after inoculation, the phenotypes of inoculated wheat seedlings were assessed and the *TaERFL1a* transcript levels were measured by qPCR. Subsequently, wheat seedlings inoculated with BSMV-VIGS-TaERFL1a and BSMV-VIGS-GFP transcript virus were separately transferred to full-strength Hoagland solution with 20% PEG6000 for 3 days. The fresh weights, dry weights, absolute water contents, and MDA concentrations of the wheat seedlings were determined as described previously [44].

### 3.5. Subcellular Localization of TaERFL1a

The coding sequence (minus the stop codon) of *TaERFL1a* was amplified and sequenced using the primers in Table S1. The subcellular localization of TaERFL1a was evaluated according to Fan et al. [45]. The verified fragments were inserted into the pCAMBIA1301 vector, which contains the 35S promoter, *GFP*, and *Kpn* I and *Bam*H I restriction sites. The recombinant pCAMBIA1301-TaERFL1a-GFP fusion vectors were transformed into the leaves of tobacco seedlings. The empty pCAMBIA1301-GFP fusion vector was used as the control. The transformed tobacco plants were incubated at 24 °C for 2–3 days, and the GFP fluorescence signals in leaves were visualized by confocal laser-scanning microscopy (LSM710, Karl Zeiss, Jena, Germany).

### 3.6. Transcription Activation of, and Proteins Interacting with TaERFL1a

A wheat leaf cDNA library was generated using the Make Your Own Mate & Plate Library System (Clontech, Mountain View, CA, USA) following the manufacturer’s instructions. The coding sequence of *TaERFL1a* was cloned into the pGBKT7 vector and transformed into the yeast strain Y2H Gold according to the manufacturer’s instructions (Clontech). AbA (0, 100, 150, and 200 ng/mL) was added to SD/–Trp medium to evaluate TaERFL1a autoactivation. AbA (100 ng/mL) was used to assay the transcriptional activity of TaERFL1a in SD/–Trp medium containing X-α-gal. An AbA concentration of 200 ng/mL, at which the autoactivation of TaERFL1a is inhibited, was used to screen the cDNA library on SD/–Trp–Leu or SD/–Trp–Leu–His–Ade medium and identify proteins interacting with TaERFL1a. The co-transformation assay was performed as described by Ramalingam et al. [46]. The TaSGT1 and TaDAD2 proteins that interacted with TaERFL1a were separately subcloned into the pGADT7 prey vector. The interaction of TaSGT1 and/or TaDAD2 with TaERFL1a was confirmed by co-transforming Y2H Gold with the bait (TaERFL1a in pGBKT7) vector, respectively. The pGBKT7-p53 and pGBKT7-Lam bait plasmids were co-transformed into Y2H Gold with the prey plasmid (pGADT7-T) as positive and negative controls, respectively. Subcellular localization of TaSGT1 and TaDAD2 proteins were analyzed by using the above described method. The interactions of TaSGT1 or TaDAD2 with TaERFL1a were also verified using by BiFC method as described by Su and his colleagues [47]. The used primers and amplified lengths are listed in Table S1. The transient assay was performed by epidermal cells of tobacco leaves, and YFP fluorescence was observed with confocal laser scanning microscopy (LSM710, Karl Zeiss, Jena, Germany).

### 3.7. Transcript Levels of the Genes Encoding Six Identified Proteins

At 3 days after the above abiotic stresses (cold, salt, and water deficiency), the uppermost fully expanded leaves of wheat plants were harvested. At 8 days after the above BSMV-VIGS experiment, the uppermost fully expanded leaves of BSMV-VIGS-TaERFL1a- and BSMV-VIGS-GFP-inoculated wheat plants were also sampled. Transcript levels of the genes encoding the six identified proteins were determined in these two experiments by using the above described qPCR method. The used primers and amplified lengths are listed in Table S1.

### 3.8. Statistical Analysis

All experiments involved at least three biological replicates, each of at least three plants. Data were subjected to one-way analysis of variance (ANOVA) using SPSS version 17.0 software (SPSS Inc., Chicago, IL, USA). Standard deviation (SD) values are from at least three biological replicates, and the significance of differences was evaluated at the *p* < 0.05 level.

## 4. Conclusions

TaERFL1a is a novel, nuclear-localized member of the B1 subgroup of the AP2/ERF superfamily that functions in the responses to multiple abiotic stresses and plays an important role in the response of wheat to water deficiency. We identified six interacting proteins of TaERFL1a, including three stress-related proteins, by yeast two-hybrid screening. Of these, two proteins (TaSGT1 and TaDAD2) were further confirmed to interact with TaERFL1a by using subcellular localization and BiFC assay. Taken together, our data suggest that TaERFL1a is a stress-responsive transcription factor, interacts with other functional proteins or regulators, and plays important and positive roles in the responses of plants to water deficiency.

## Figures and Tables

**Table 1:** Effect of TaERFL1a silencing on some growth and physiological parameters of wheat seedlings suffering from water deficiency.

Treatments	Fresh Weights (g/Plant)	Dry Weights (g/Plant)	Absolute Water Contents (%)	MDA Concentrations (nmol·L^−1^ FW)
BSMV-GFP	0.49 ± 0.024 ^a^	0.08 ± 0.007 ^a^	84.42 ± 0.650 ^a^	18.98 ± 1.144 ^a^
BSMV-TaERFL1a	0.22 ± 0.006 ^b^	0.06 ± 0.020 ^b^	74.56 ± 0.912 ^b^	31.80 ± 0.639 ^b^

Each value is mean ± standard deviation of three independent biological replicates. Different letters represent statistical significance at *p* < 0.05.

**Table 2:** The identified protein species with interaction with TaERFL1a protein via yeast two-hybrid screening.

No.	Homology Accession No.	Homologous Protein	Plant Species	Clone Numbers	Homology
1	JQ269674	Cu/Zn Superoxide dismutase	*Triticum aestivum*	1	97.8%
2	KJ907387.1	SGT1	*Triticum aestivum*	1	99.3%
3	GU564291	Defender against cell death 2	*Triticum aestivum*	1	100.0%
4	XM_020309713	Pyrrolidone-carboxylate peptidase	*Aegilops tauschii*	1	85.6%
5, 6, 7	AM087943	Avenin-like a precursor	*Aegilops tauschii*	3	93.2%
8, 9, 10	XM_020326125	MOB kinase activator-like	*Aegilops tauschii*	3	95.3%

## Supplementary Materials

The following are available online at http://www.mdpi.com/1422-0067/19/5/1465/s1.
