# Supplementary material for: Function of the ERFL1a Transcription Factor in Wheat Responses to Water Deficiency
Source: Int J Mol Sci. 2018 May 15;19(5):1465. doi: 10.3390/ijms19051465 (PMC5983727; doi:10.3390/ijms19051465)
Supplement: Supplementary file 1 [file ijms-19-01465-s001.pdf]

```
BQ789086   ...........................................................................
TaERFL1a   TACGCGCACGCCACTTGTACACCTGCTGCACCCATGGCGCCTAGAGCGGCGGAGAAGGCGCCTGTCTCCCCGCCC   75

BQ789086   ...........................................................................
TaERFL1a   ACCAGGCTCGGCCTTGGCGTTGGCGGCGGGGTCGGAGTCGTCGCCGGTGGCGCGCACTACAGGGGCGTCCGGAAG  150

BQ789086   ...........................................................................
TaERFL1a   CGCCCATGGGGACGTTACGCCGCGGAGATCCGTGACCCGGCCAAGAAGAGCAGGGTCTGGCTCGGCACGTACGAT  225

BQ789086   ...........................................................................
TaERFL1a   ACGGCGGAGGAGGCCGCGCGCGCCTACGACACCGCCGCGCGCGAGTTCCGCGGCGCCAAGGCGAAAACTAACTTC  300

BQ789086   ...........................................................................
TaERFL1a   CCGTTCCCTTCGTCGTCGTCGTCGTCCCCTGTCGCCGGCGGCGGCAGCCCGAGCAGCAACAGCACCTTGGACTCT  375

BQ789086   GAGGGCGGAGGGAGCGGCGGCTGCGCCCAGGCGCCCATGCAGGCCATCCCGCTGCCGCCCGCCCTCGACCTGGAC   75
TaERFL1a   AGCGGGGGAGGGAGCGGCGGCTGCGCCCAGGCGCCCATGCAGGCCATCCCGCTGCCGCCCGCCCTCGACCTGGAC  450

BQ789086   CTCTTCCACCGCGCGGCGGTCGTCACGGCCGTCGCCGGCGGCGGCATGCGCTTCCCATTCAACGGTTACCCGGTG  150
TaERFL1a   CTCTTCCACCGCGCGGCGGCCGTGACGGCCGTCGCCGGCGGCGGCATGCGCTTCCCGTTCAACGGTTACCCGGTG  525

BQ789086   GCGCCGCGCCAGCCCCTGCACCCGTACTTCTTCTACGAACAGGCCGCGGCCGCCGCGGCGGCGTCGGCGTCGGGG  225
TaERFL1a   GCATCGCGCCAGCCTCTGCACCCGTACTTCTTCTACGAACAGGCCGCGGCCGCCGCGGCGG...CGGCGTCGGGG  597

BQ789086   TACCGCGCGCTGAAGGTGGCGCAGCCGGTCACCGTGGCGGCCGTTGCGCAGAGCGACTCCGACTCCTCGTCGGTC  300
TaERFL1a   TATCGCGCGCTGAAGGTGGCGCAGCCGGTCACCGTGGCGGCCGTTGCCCAGAGCGACTCCGACTCCTCGTCGGTC  672

BQ789086   GTTGATCTGTCCCCGTCGCCCCCCGCGGTGACAGCGCATAAGGCGGTCGCGTTTGATCTGGATCTCAACCGGCCG  375
TaERFL1a   GTTGATCTGTCCCCGTCGCCCCCCGCGGTGACAGCGCATAAGGCGGTCACGTTTGATCTGGATCTGAACGGGCCG  747

BQ789086   CCGCCTTCCGAGGACTAGACAAAGGACAAATTTTAGATGATGACTGTAGTTTCTCATTTTTCCTGCAGGGAACGC  450
TaERFL1a   CCGCCTTCGGAGGACTAGACAAAGGACAAATTTTAGATGATGACTGTAGTTTCTCATTTTTCCTGCAGGCAACT.  821

BQ789086   TTTTTTTCCTTCTTCTGTTCTGGTCCTCTTGTATTTTTGTTTAGTTGTCCGAGACAGACCGAGGAGCCCTGTAAA  525
TaERFL1a   TTTTTTTCCTTCTTCTGTTCTGGTCCTCTTGTATTTTTGTTTAGTTGTCTGAGACAGACCGAGGAGCC.......  889

BQ789086   TAGTTTTTCCGCCGAGAACAGAGCAGAACCGATCTGAGATCTGTTGGTCTAAAACGGATCAAACCGGCGCTGAGG  600
TaERFL1a   ...........................................................................

BQ789086   TGAACCGAGACTTGAGTATATGTGTACTCCTCATAAGATGATGAATTTTTCAGAAGATTATGTCCATCAAAAAAA  675
TaERFL1a   ...........................................................................

BQ789086   AAAAAAAAAAAAAAAAAAA                                                          694
TaERFL1a   ...................
```

Figure S1. Comparison between the downloaded EST and the amplified nucleic acid sequences of *TaERFL1a* gene. EST sequence (GeneBank: BQ789086) of *TaERFL1a* is derived from NCBI database.

```
TaERFL1a-3AL ATGGCGCCTAGAGCGGCGGAGAAGGCGCCTGTCTCCCCGCCCACCGGCCTCGGCCTCGGCGTCGGCGGCGGGATC  75
TaERFL1a-3B  ATGGCGCCTAGAGCGGCGGAGAAGGCGCCTGTCTCCCCGCCCACCAGGCTCGGCCTTGGCGTTGGCGGCGGGGTC  75
TaERFL1a-3DL ATGGCGCCTAGAGCGGCGGAGAAGGCGCCTGTCTCCCCGCCCACCGGGCTCGGCCTCGGCCTCGGCGGCGGGGTC  75

TaERFL1a-3AL GGGGTCGTCGCCGGCGGCGCGCACTACAGGGGCGTCCGGAAGCGCCCGTGGGGACGCTACGCCGCGGAGATCCGC 150
TaERFL1a-3B  GGAGTCGTCGCCGGTGGCGCGCACTACAGGGGCGTCCGGAAGCGCCCATGGGGACGTTACGCCGCGGAGATCCGT 150
TaERFL1a-3DL GGGGTCGTCGCCGGGGGCGCGCACTACAGGGGCGTCCGCAAGCGCCCGTGGGGACGTTACGCCGCGGAGATCCGT 150

TaERFL1a-3AL GACCCGGCCAAGAAGAGCAGGGTGTGGCTCGGCACGTACGACACCGCGGAGGAGGCCGCGCGCGCCTACGACACC 225
TaERFL1a-3B  GACCCGGCCAAGAAGAGCAGGGTCTGGCTCGGCACGTACGATACGGCGGAGGAGGCCGCGCGCGCCTACGACACC 225
TaERFL1a-3DL GACCCGGCCAAGAAGAGCAGGGTGTGGCTCGGCACGTACGACACGGCGGAGGAGGCCGCGCGCGCCTACGACACC 225

TaERFL1a-3AL GCCGCCCGCGAGTTCCGCGGCGCCAAGGCGAAGACCAACTTCCCGTTCCCTTCGTCGTCGTTGTCCCCTGTCGCC 300
TaERFL1a-3B  GCCGCGCGCGAGTTCCGCGGCGCCAAGGCGAAAACTAACTTCCCGTTCCCTTCGTCGTCGTCGTCGTCCCCTGTC 300
TaERFL1a-3DL GCCGCGCGTGAGTTCCGCGGCGCCAAGGCGAAAACCAACTTCCCGTTCCCTTCGTCGTCGTCGTCGTCCCCTGTC 300

TaERFL1a-3AL GCCGCCGGCGGCGGCGGCGGCAGCCCGAGCAGCGACAGCACCTTGGACTCGAGCGGCGGAGGGAGCGGCGGCTGC 375
TaERFL1a-3B  GCCG.........GCGGCGGCAGCCCGAGCAGCAACAGCACCTTGGACTCTAGCGGGGGAGGGAGCGGCGGCTGC 366
TaERFL1a-3DL GCCGGCGCCGGCGGCGGCGGCAGCCCGAGCAGCGACAGCACCTTGGACTCGAGCGGCGGAGGGAGCGGCGGCTGC 375

TaERFL1a-3AL GCCCAGGCGCCCATGCAGGCCATCCCGCTGCCGCCCGCCCTCGACCTGGACCTCTTCCACCGCGCGGCGGTCGTC 450
TaERFL1a-3B  GCCCAGGCGCCCATGCAGGCCATCCCGCTGCCGCCCGCCCTCGACCTGGACCTCTTCCACCGCGCGGCGGCCGTG 441
TaERFL1a-3DL GCCCAGGCGCCCATGCAGGCCATCCCGCTGCCGCCCGCCCTCGACCTGGACCTCTTCCACCGCGCGGCGGCCGTC 450

TaERFL1a-3AL ACGGCCGTCGCCGGCGGCGGCATGCGCTTCCCATTCAACGGTTACCCGGTGGCGCCGCGCCAGCCCCTGCACCCG 525
TaERFL1a-3B  ACGGCCGTCGCCGGCGGCGGCATGCGCTTCCCGTTCAACGGTTACCCGGTGGCATCGCGCCAGCCTCTGCACCCG 516
TaERFL1a-3DL ACGGCGGTCGCCGGCGGCGGCATGCGCTTCCCATTCAACGGTTACCCGGTGGCGCCGCGCCAGCCCATGCACCCG 525

TaERFL1a-3AL TACTTCTTCTACGAACAGGCCGCGGCCGCCGCGGCGGCGTCGGCGTCGGGGTACCGCGCGCTGAAGGTGGCGCAG 600
TaERFL1a-3B  TACTTCTTCTACGAACAGGCCGCGGCCGCCGCGGCGGC...GGCGTCGGGGTATCGCGCGCTGAAGGTGGCGCAG 588
TaERFL1a-3DL TACTTCTTCTACGAACAGGCCGCAGCGGCGGCGGCGGC...GGCGTCGGGGTACCGCGCGCTGAAGGTGGCGCAG 597

TaERFL1a-3AL CCGGTCACCGTGGCGGCCGTTGCGCAGAGCGACTCCGACTCCTCGTCGGTCGTTGATCTGTCCCCGTCGCCCCCC 675
TaERFL1a-3B  CCGGTCACCGTGGCGGCCGTTGCCCAGAGCGACTCCGACTCCTCGTCGGTCGTTGATCTGTCCCCGTCGCCCCCC 663
TaERFL1a-3AL CCGGTCACCGTGGCGGCCGTTGCTCGGAGCGACTCCGACTCCTCGTCGGTCGTTGATCTGTCCCCGTCGCCCCCC 672

TaERFL1a-3AL GCGGTGACAGCGCATAAGGCGGTCGCGTTTGATCTGGATCTCAACCGGCCGCCGCCTTCCGAGGACTAG       744
TaERFL1a-3B  GCGGTGACAGCGCATAAGGCGGTCACGTTTGATCTGGATCTGAACGGGCCGCCGCCTTCGGAGGACTAG       732
TaERFL1a-3AL GCGGTGACAGCGCATAAGGCGGTCGCGTTTGATCTGGATCTGAACCGGCCGCCGCCTTCCGAGGACTAG       741
```

Figure S2. The coding sequences for three *TaERFL1a* copies derived from IWGSC database. The AP2/ERF DNA binding domains of ERF proteins are signed with single dotted line. The fragment used to construct BSMV-VIGS-TaERFL1a vector is signed with single solid line. The black color is defined to 100% similarity among three copies.

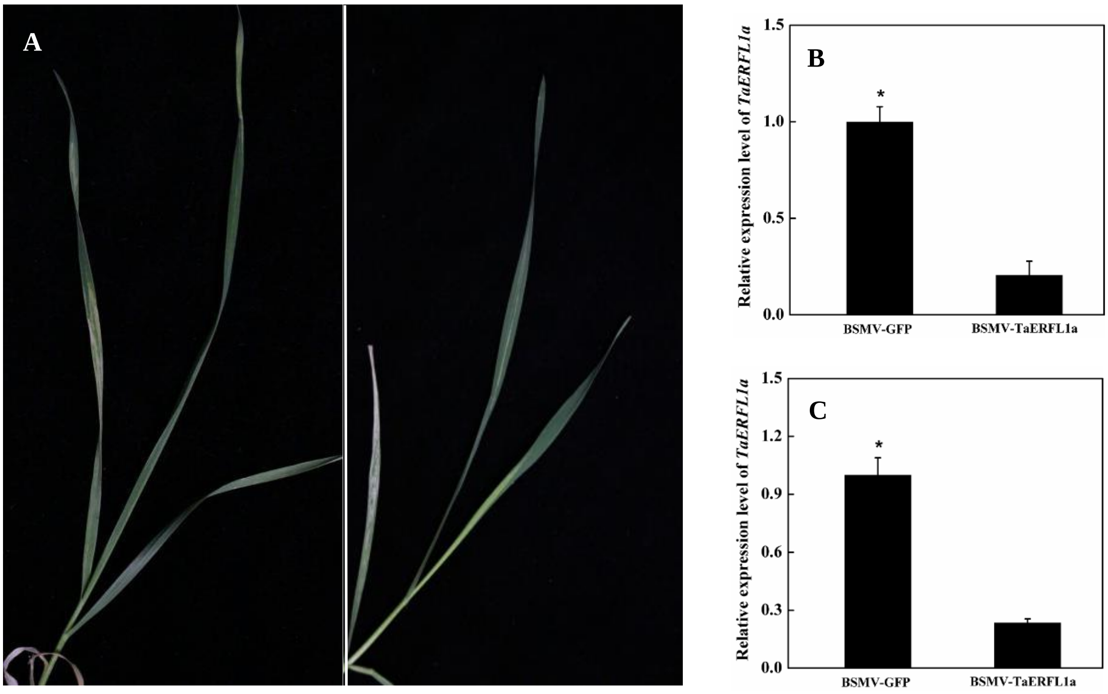

BSMV-GFP **BSMV-TaERFL1a**

Figure S3. Phenotypes (A) and transcript levels of *TaERFL1a* (B and C) on the BSMV-VIGS-TaERFL1a-inoculated- or BSMV-VIGS-GFP-inoculated wheat seedlings at 8 d after virus inoculation. Phenotypes of BSMV-VIGS-TaERFL1a-inoculated and BSMV-VIGS-GFP-inoculated wheat seedlings are observed at 8 d after virus inoculation. Transcript levels are determined by qPCR method using *Actin* (B) and *GAPDH* (C) genes as internal controls. Each value is mean ± standard deviation of three biological replicates. Asterisks indicate significant differences ($p < 0.05$).

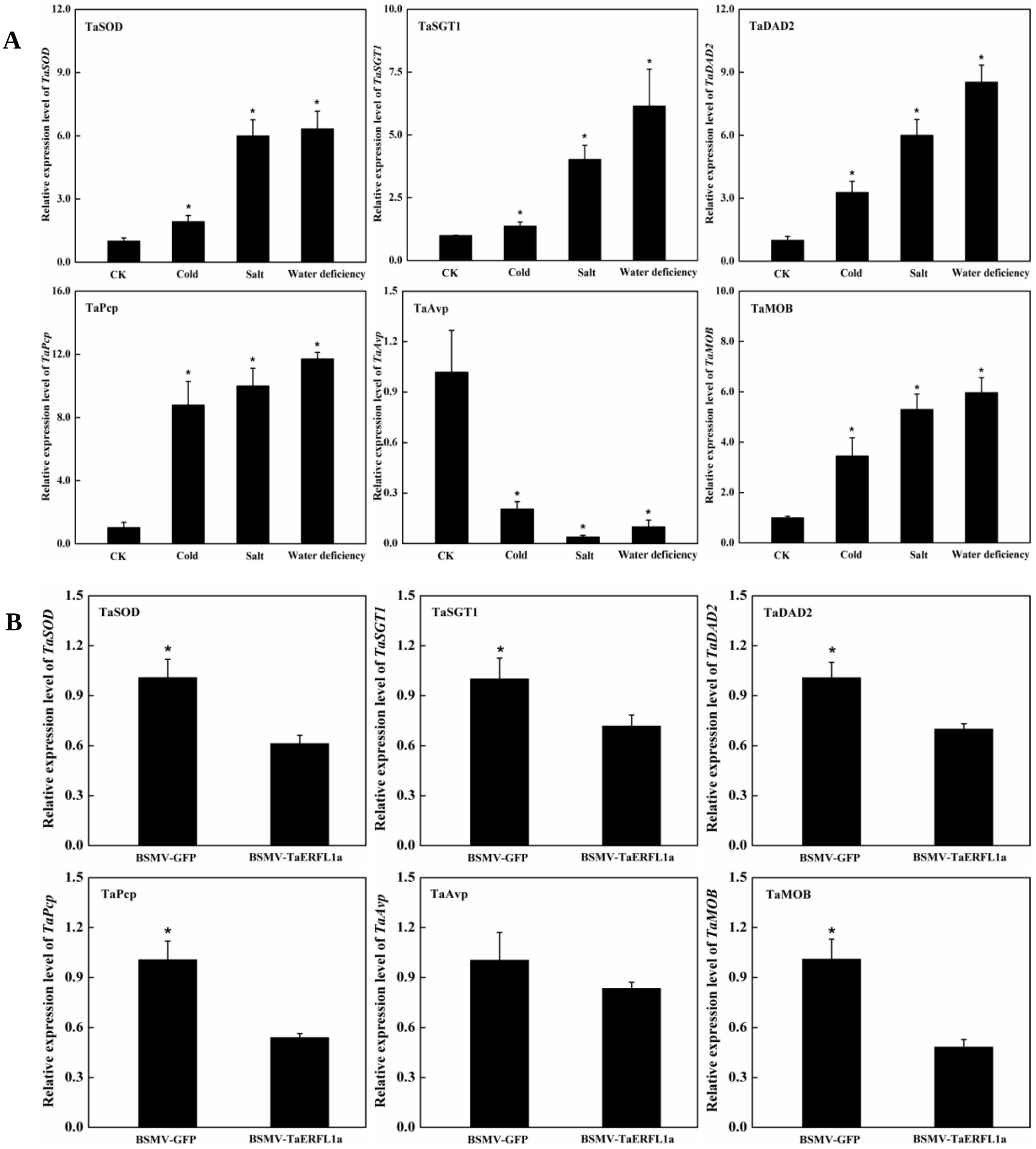

Figure S4. Transcript levels of the genes encoding six identified proteins in leaves of wheat plants suffering from cold (4°C), salt (250 mM NaCl), and water deficiency (20% PEG6000) for 3 d (A), and BSMV-VIGS-TaERFL1a-inoculated- or BSMV-VIGS-GFP- inoculated wheat seedlings at 8 d after virus inoculation (B). TaSOD, Cu/Zn SOD; TaPcp, a pyrrolidone-carboxylate peptidase; TaAvp, an avenin-like a precursor; TaMOB, an MOB kinase activator-like. Transcript levels are determined by qPCR using *Actin* gene as internal control. Each value is mean ± standard deviation of three biological replicates. Asterisks indicate significant differences ($p < 0.05$).

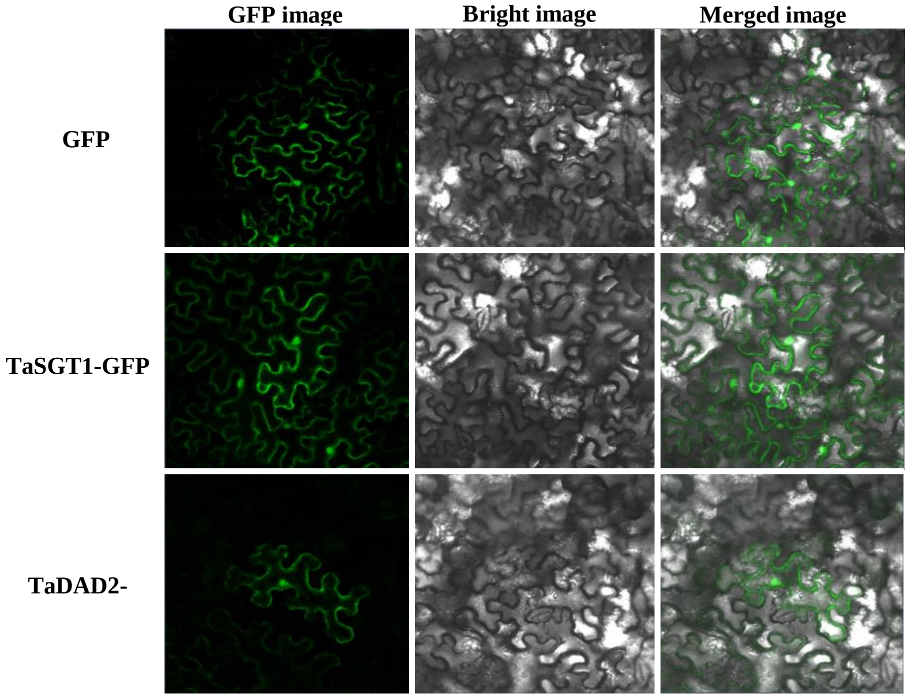

Figure S5. Subcellular localization of TaSGT1 and TaDAD2 proteins in tobacco leaves. GFP, subcellular localization of single GFP in tobacco leaves; TaSGT1-GFP, subcellular localization of TaSGT1-GFP fusion protein in tobacco leaves; TaDAD2-GFP, TaDAD2-GFP fusion protein in tobacco leaves. Bar = 40 μm.

Promoter of the gene encoding the SGT1 protein

```
GAGCTATCCCCTGCTTTTGCAGGTCAATTACTCCCAGCCCGCCGAAGCACTTAGGTCTGCAAACCCTCTGCCAGGACACAACGCACTTGCCGCCAATA
ATATGATCTGAGCCGGCCCAAAAGAAAGCTCTGCAGCCCTTGTCCACCTTCTCCAATGCCCATTTGGGAGCTTGAGCCACAATCATGTGATGTGTAGG
TCTTGCCCTCATCACCTAGTTAACTAGGATCAGCCTTCCGGGCTGGGTCACCATCCCTCTCTGCCATCCCGGCAGGATTCTCAATGCAGAGTCAATCA
TTGGCTGCCAATCCGAACATTTGAGCTGTTTGATGCTGAGCTGTAAACCTAAGTATCTACATGGGAACTTCCCGATCTGCTAGGGCACCATGCCTCGG
ACCAACTCCTCATCCCCTTCTTCCCCTCTGATAATAATGGCAAACGACTTAGCGTAGTTCACCTTAAGCCCTAATGCCTCTTCGAAGATCTTGAGAAT
CTCTAGAACAAATGACAAATCCGCATAGTTTGGCTTGACGAATAAGACCACATCATCGGTGTACAGGGACAGTCTTTGCGTGGCTCCGCAACCATTCA
TTTTACTCAGTATCTGGGATTCATGTGCCTTGATGATGATGGCTGTAAGAACATCCATCGCGATGACGAACAACAAAGGCGACAGGGACTCCCCTTGC
CTCAGACCGCAAGCATGCCTGAACTTCTCCCCAGCGCATCCATTGACCAACACGCGGGAGCTGGCCGTGGAAAGAAGTGTAAAGACCGAAGATAGCCA
CCGCTTGGAGAATCCCTTTGCCCGTAGCACCTCAAAGAGAAACGGCCACGATAGGGAGTCGAAGGCCTTCGAAATGTCTAACTTGGAGCAGCACGCAT
TTAGCTTTCCTCTTATGCAGCCGCCGCGCCAATAGCCTGACCAGCAGAAAGTTGTCATGTATGCTGCGTCCTTTGATGAACGCCGACTGATTAGCGTT
GACAAGCTCAGGCATTCGCTTGCAAAGTCTGGATGCCAACAGTTTCGACGCCATTTTGGGTATGTTATGTACCAAGCAGATCGGCCTGAAGTCCTTGA
CAGTGCATGCTGCCGCTGTTTTTGGAATGAGTGAGATGAGCGCCTGGTTGAGCCTCCCGAAGCCGCGTCCATTATCCAGAAACAATTTGTGCAGGGCG
GCAAGGATATCACGCTTGATCACATTCCATGCTTTTTGAAAGAAGGCACCAATAAATCCATCTGGCCCCGTCCCAAATTCCGTCCTCCTGAAACACCA
AGTCTTGCTCTGAGAGATCCAGCAGTTGGATGTTTAGAAAGTCAAGATCAACCGTGTGCTCTCGAGCCGAGTCCTTCCCCATAATCTCCCTGAAGGTT
TCAAGGAAGGCCTCCTCCTTCCCCTTTTGATCAGTGATTGTCTGTCCGTTGCTTGAGTTATGAGGGTACCTAGCATGCGAAGATGCCATGCAGCGTTT
AGTATCATTAATCCAACATCATATTCTAATCTCCATTGACGACGCACAACTGCACAAGCCAACGACTGCACATGGCGCCGATGACAGCCTTCTTGCTC
CCTAATCACGATCTCAGACACCAAGATCGAAAAAAGTCACCCCCTTAACCCCATAGCAAGAGGCTAATCAAGCAATGATTCGTGAAACTTCATGGGTC
CCAAAAGCAAAACAATTAGGCTTGATCCGCACAAAAGACAGAAAGGAAAGGAAAGGAAAGGAAAAAGAGAGGAGGAAAAGCAAAAAACAAAGGACGGA
GAACTTGCCATGCACTGCAACTCCAATCCCCCCCTCGGCCTGCTGGCCGGCCCTCCCCCCGGAACACTCTAGAAACAGCCCCACGGGCCCTTAAGAGG
CCGTTTAACCGCACGCAGCTCAATCCAATCCAATCAGTCCGTTGCTTGGATCCATCCATCCCCTCCCCACCAAATACCGTCGATCTCTATTCACCTCT
CCCAAAGTCTTCGTCTGGTCTTCTCCACCTCGACGCAGAC
```

Figure S6. Promoter sequence of the gene encoding the SGT1 protein identified in the Y2H experiment. Promoter sequence is derived from IWGSC database. The GCC-box and DRE/CRT elements are signed with boxes and single solid lines, respectively.

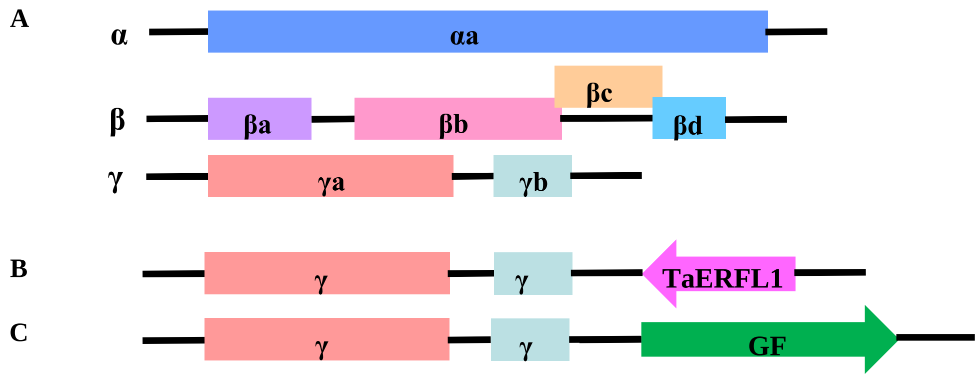

Figure S7. Schematic diagrams of BSMV-VIGS-TaERFL1a and BSMV-VIGS-GFP vectors. A, Diagrams of BSMV virus; B and C, Schematic diagrams of BSMV-VIGS-TaERFL1a and BSMV-VIGS-GFP vectors, respectively.

Table S1. The primer sequences used in this study.

Genes	Primer sequences (5′-3′)	Usage	Amplification sizes (bp)
TaERFL1a	1F: TACGCGCACGCCACTTGTACACC<br>1R: AGCCCTGTAAATAGTTTTTCCGCCG	Amplification of cDNA sequence for TaERFL1a	981
	2F: CCTTAATTAAGCGGCGGCATGCG<br>2R: TATGCGGCCGCCAACGACCGACGAG	Construction of BSMV-VIGS-TaERFL1a vector	249
	3F: CTTCCCGGGATGGCGCCTAGAGCGGC<br>3R: CAGGTCGACGTCCTCCGAAGGCGGCG	Construction of YN-TaERFL1a vector	745
	4F: CGGGGTACCATGGCGCCTAGAGCGGCGGAG<br>4R: CGCGGATCCGTCCTCCGAAGGCGGCGGCCCG	Subcellular location	741
	5F: CGGAATTCATGGCGCCTAGAGCGGCGGA<br>5R :AAACTGCAGCTAGTCATCCGAAGGCGGCG	Transcription activation assay	741
	6F: GCCAAGGACCTCGTTAGAAAGA<br>6R: GGCCCGTTCAGATCCAGAT	qPCR	154
TaSGT1	F: GTACCCGGGATGGCCGCCGCCGCCGC<br>R: ACATACTAGTATACTCCCACTTCTTGAG	Construction of YC-TaSGT1 vector	1132
	F: ATGCATCTGGTGACTCGAGG<br>R: AGTTGTAGCCCCGGAAGATG	qPCR	146
TaDAD2	F: GGTACCCGGGATGCCGAAGCCCGCGGG<br>R: ACATACTAGTTCCGAGGAAGTTCATGG	Construction of YC-TaDAD2 vector	345
	F: GGTGTCCTGTCATGCATTGG<br>R: GCACAGGACGAAATCAGCAA	qPCR	119
TaSOD	F: GCTAGCCAGGTGCCAGTAAA<br>R: TACCGTTGGCTGTGCATTCT	qPCR	137
TaMOB	F: AATTCTCAAAACAGCCGGGC<br>R: ATTTGCCCCTGAGCTGATGA	qPCR	164
TaPcp	F: GAGAACGGGTCATCAGCTCA<br>R: TTGTCCGTGAGATGCTTCCA	qPCR	184
TaAvp	F: GCAGGTTCCGGTCGAGATAA<br>R: ATGGGGATGCTGTAGATGGC	qPCR	122
Actin	F: AAACGAAGGATAGCATGAGGAAGC<br>R: AGCGGTCGAACAACTGGTA	qPCR	101
GAPDH	F: TTTTCACCGACAAGGACA<br>R: AAGAGGAGCAAGGCAGTT	qPCR	179
